# Herpes Simplex Virus Type 1-Induced Cervicitis Without Vulvar Lesions Mimicking Cervical Cancer in a Healthy Woman

**DOI:** 10.7759/cureus.78128

**Published:** 2025-01-28

**Authors:** Akina Nigi, Tomohisa Kihira, Akinobu Hayashi, Tadashi Yabana, Hiroyuki Tanaka

**Affiliations:** 1 Obstetrics and Gynecology, Japanese Red Cross Ise Hospital, Ise, JPN; 2 Pathology, Japanese Red Cross Ise Hospital, Ise, JPN; 3 Infectious Diseases, Japanese Red Cross Ise Hospital, Ise, JPN

**Keywords:** cervical cancer, cervical swelling, cervicitis, genital infection, herpes simplex virus, hsv, perinatal complication

## Abstract

Genital herpes, caused by herpes simplex virus (HSV) types 1 and 2, is a common sexually transmitted disease typically presenting with genital lesions and discomfort. However, a cervical mass without vulvar lesions is a rare manifestation of the disease, often leading to diagnostic confusion with cervical cancer. This report highlights a unique case of HSV type 1-induced cervical swelling in the absence of vulvar lesions, confirmed through pathological diagnosis. To our knowledge, this is the first documented instance where HSV type 1-induced cervical mass was confirmed through pathological diagnosis. Magnetic resonance imaging, pathological analysis, and immunohistochemistry were key in distinguishing the condition from malignancy. This case underscores the importance of considering HSV infection in differential diagnoses for a cervical mass and provides critical insights for the accurate diagnosis and management of HSV-related cervicitis.

## Introduction

Genital herpes is a very common sexually transmitted disease that affects an estimated half a billion people, especially sexually active people [1]. The main pathogenic microorganisms are herpes simplex virus (HSV) types 1 and 2, which have the ability of immune evasion and residence in the cell nucleus as an episome, causing the possibility of life-long infection and reactivation [2]. Major symptoms of genital herpes include one or more small papules, vesicles, or vulvar ulcers with pain; however, it can sometimes remain asymptomatic [3]. Genital herpes rarely causes cervical swelling, with only three reported cases to our knowledge [4-6]. Gynecologists are likely to initially suspect cervical cancer as the cause of cervical swelling, as several malignant tumors of the cervix can present with similar symptoms. These include cervical adenocarcinoma, minimal deviation adenocarcinoma, and lobular endocervical glandular hyperplasia. However, other potential causes of cervical swelling or masses should also be considered in the differential diagnosis. These include benign conditions such as cervical polyps, fibroids (leiomyomas), endometriosis, and Nabothian cysts. Additionally, infectious causes, such as chronic cervicitis or pelvic inflammatory disease, as well as HSV infections, must be evaluated. Less common conditions, such as lymphoma or sarcoma, should also be considered when investigating cervical masses [7]. Therefore, it is very difficult to differentiate genital herpes from cervical cancer in a case of cervical swelling. In this case, to our knowledge, we introduce the first case of HSV type 1-induced cervical swelling diagnosed pathologically.

## Case presentation

A 37-year-old female without any past medical or medication history presented with abound watery discharge with more subtle bleeding than usual, dysuria, abdominal pain, and high-grade fever. Her obstetric history included two normal vaginal deliveries at full term. Her spouse was diagnosed with genital HSV infection from another sexual partner three years ago, presenting with small papular genital lesions. However, as the patient was asymptomatic, she was not screened for HSV at that time, and no therapy was provided to her. On day five after the onset of symptoms, she visited a gynecologist. Although oral tosufloxacin was administrated as an empirical therapy for urinary tract infection, fever was not relieved. The discharge was clear with no malodor, and polymerase chain reaction (PCR) tests of vaginal secretion for *Chlamydia trachomatis* and *Neisseria gonorrhoeae* were negative. Serological tests for *Treponema pallidum* and human immunodeficiency virus (HIV) were also negative. On day 10, she visited the previous practitioner again without improvement of symptoms. In addition to a large quantity of clear vaginal discharge, vaginal ultrasonography showed the enlargement of the cervix. On day 11, she was referred to our hospital for further examination and treatment due to a high suspicion of cervical cancer.

**Figure 1:**
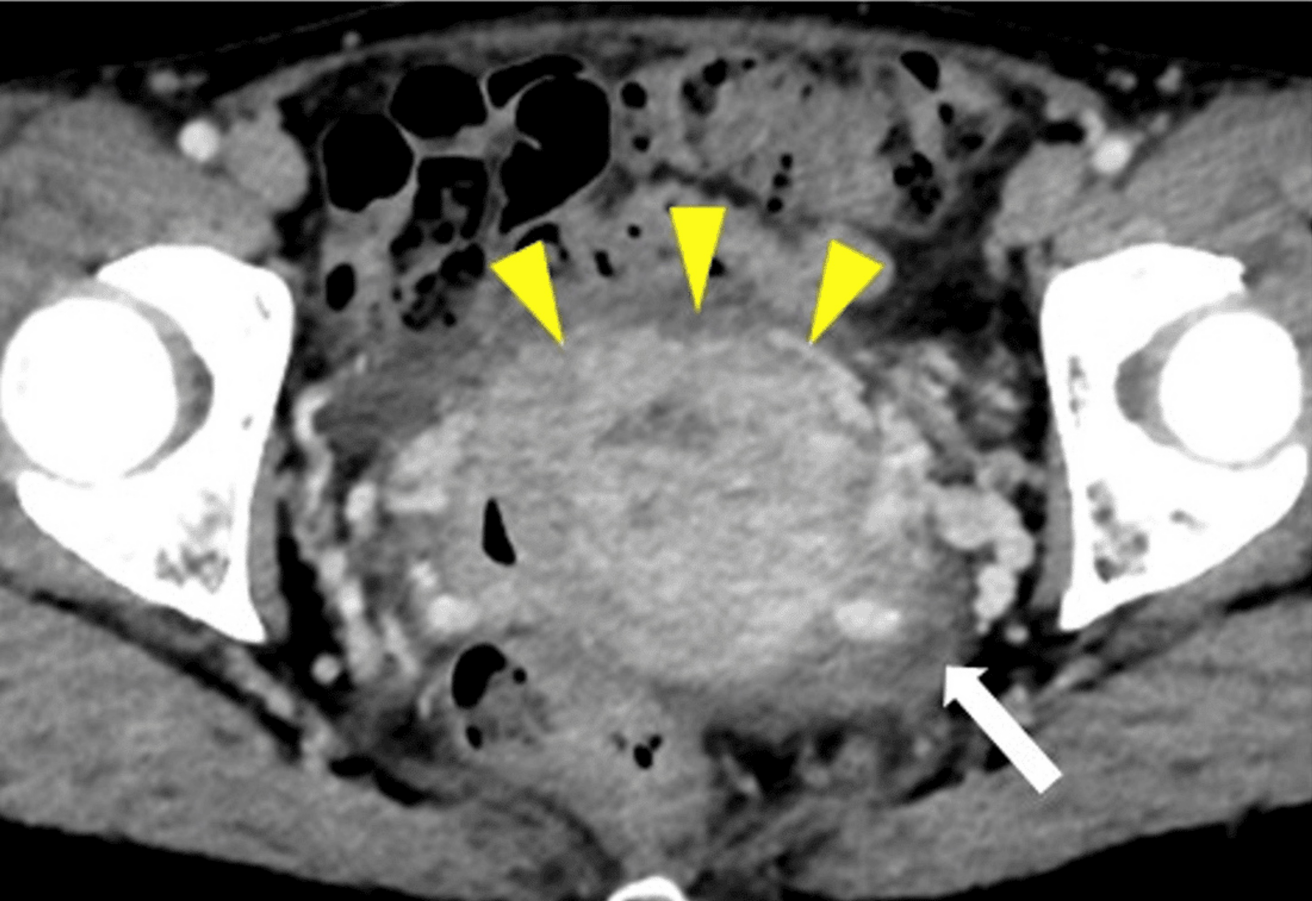
Computed tomography (CT) findings. The contrast-enhanced CT shows cervical swelling. The yellow arrowhead indicates cervical swelling. The white arrow indicates lymph node enlargement.

**Figure 2:**
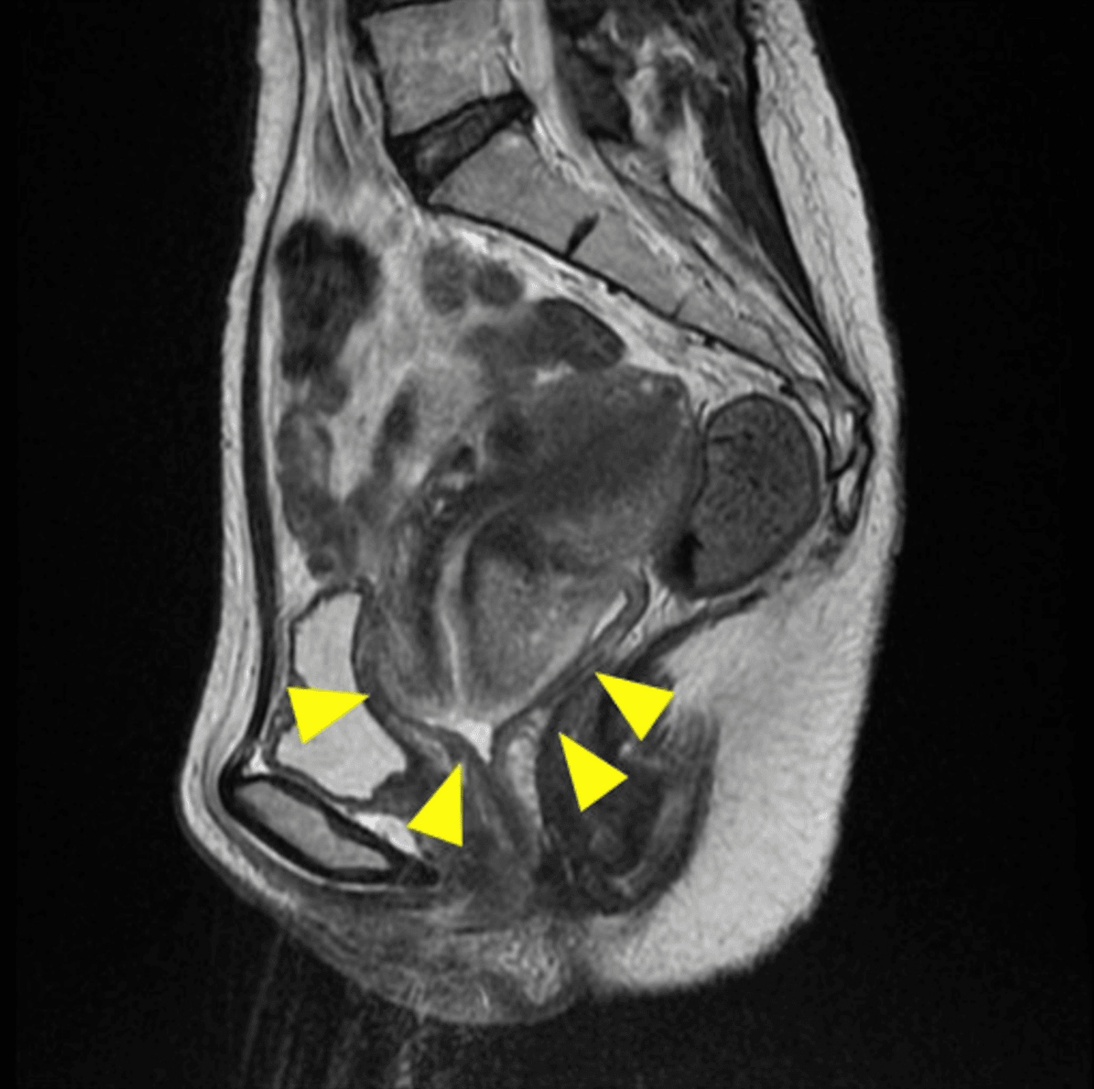
Magnetic resonance imaging (MRI) findings. MRI showing edematous cervical swelling with a preserved junctional zone (yellow arrowheads), without any findings suggestive of malignancy.

Her vital signs on admission were a body temperature of 38℃, blood pressure of 118/70 mmHg, pulse rate of 95 beats/minute, and oxygen saturation of 98% on room air. There were no abdominal findings, skin lesions, and lymphadenopathy. On pelvic examination, the patient presented with a significant amount of clear vaginal discharge and mild bleeding. No genital ulcers were observed. Upon inspection of the cervix, a large, swollen mass was noted. The swollen cervical mass measured approximately 5.5 cm in diameter. The mass was slightly soft to palpation and tender upon pressure. There were no signs of friability or active bleeding. Despite the swelling, the cervical surface appeared intact, without any visible erosions or ulcerations. Pelvic computed tomography (CT) showed remarkable cervical swelling with a small amount of ascites (Figure 1). Although intravenous administration of ceftriaxone and azithromycin was initiated for suspected genital infection caused by *Chlamydia trachomatis* or *Neisseria gonorrhoeae* on admission, it was discontinued due to no response on day 10. Based on CT findings and the ineffectiveness of antibiotics, the attending gynecologist initially planned to perform a total hysterectomy with extensive dissection for suspected cervical cancer before magnetic resonance imaging (MRI) was conducted. However, MRI revealed a preserved junctional zone in the thickened cervix, and tumor invasion was not clarified (Figure 2). Furthermore, the cytosmear test obtained by scratching the cervical lesion gently with a brush and staining with Papanicolaou stain showed that ground glassy multinucleated giant cells, showing margination of nuclei, were present in the cervical squamous epithelium. Cervical cytology indicated the possibility of HSV infection.

**Figure 3:**
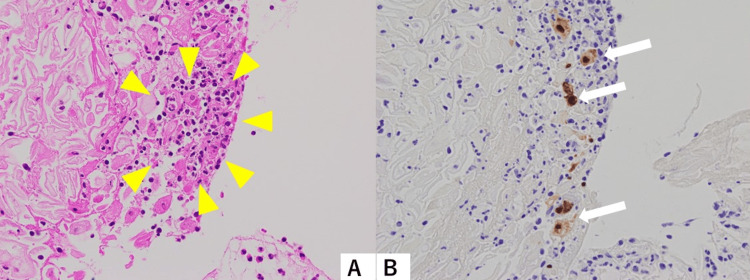
Histopathology findings. (A) Hematoxylin and eosin-stained histopathological specimen shows granulations with erosion and necrosis (yellow arrowheads) at 400× magnification. (B) Immunohistochemical staining with the antibody to herpes simplex virus type 1 (HSV-1) depicts cervical epithelial cells with nucleomegaly (white arrows) at 400× magnification. The sections were stained with a polyclonal antibody against HSV-1 (GeneTex,Cat#36599).

**Figure 4:**
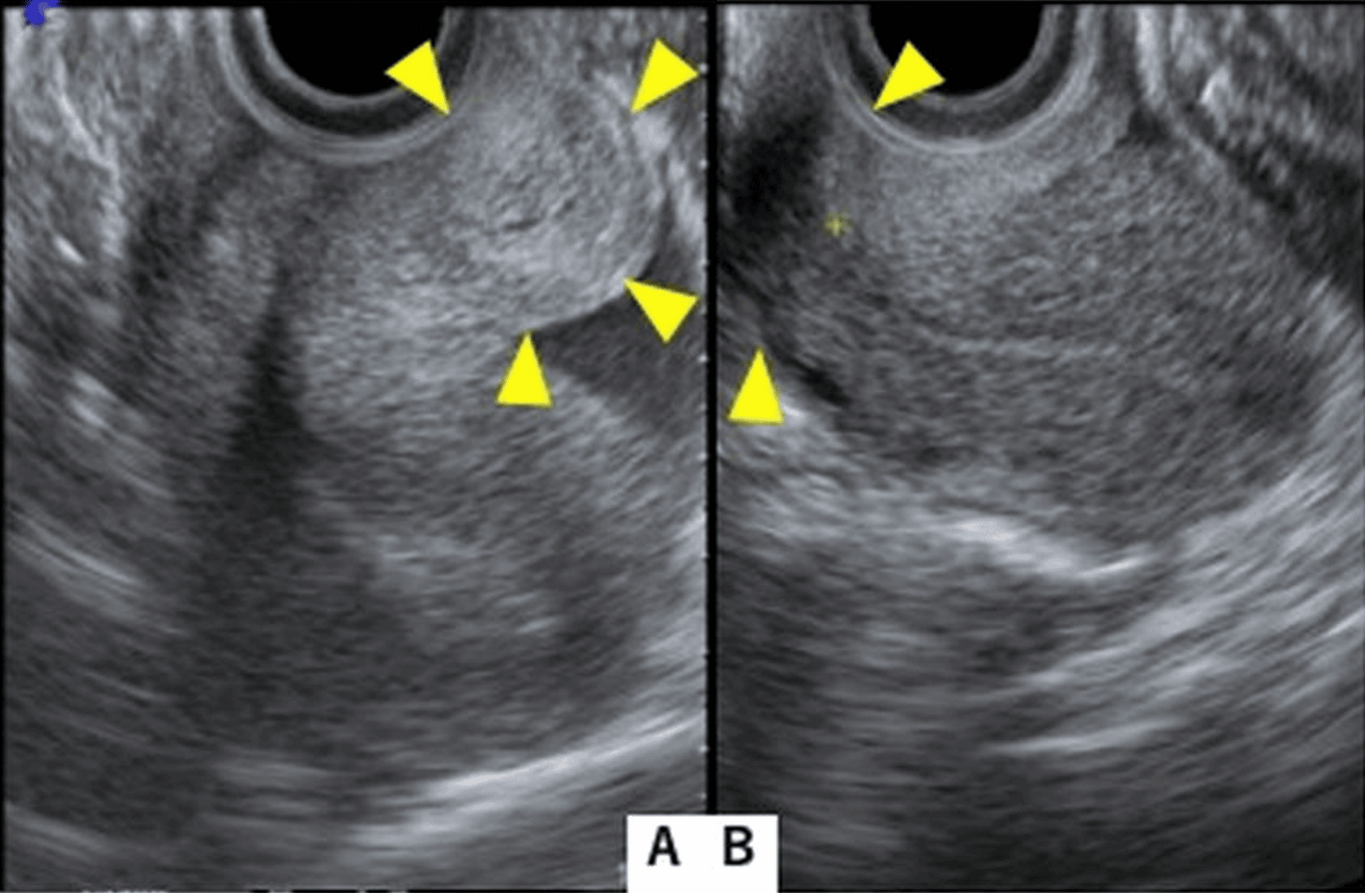
Ultrasonography findings. (A) Vaginal ultrasonography at the first visit shows swelling of the cervix (yellow arrowheads). (B) Vaginal ultrasonography performed after three months shows normal findings of the cervix (yellow arrowheads).

A punch biopsy was performed as an incision biopsy to obtain a tissue sample for pathological analysis. The pathological examination with immunohistochemistry (IHC) staining revealed that the tissues were composed of granulomatous lesions with erosion and necrosis (Figure 3). After MRI and further pathological examinations, including biopsy, it was determined that the cervical swelling was caused by HSV-related cervicitis. Consequently, the planned surgery was not performed. On day 21, symptoms spontaneously improved without specific treatment, such as the use of antiviral drugs. On day 90, the Pap smear test was negative, and vaginal ultrasonography showed a normal-sized cervix (Figure 4). One year after onset, symptoms have not recurred.

## Discussion

In this case, a gynecologist initially assumed that a malignant tumor of the cervix caused cervical swelling as most cases of malignant tumors cause a bulky mass and CT could not rule out cervical cancer. Typically, cervical cancer is asymptomatic in the early stages and presents with abnormal vaginal bleeding, post-coital pain, limb edema, flank pain, and sciatica in the advanced stages [8]. If hydronephrosis due to the tumor invasion of ureters causes urinary tract infection, fever or lower back pain may occur. This case did not have these symptoms; therefore, alternative diagnoses should have been considered on admission, such as uterine pyometra, myoma of the cervix, or genital infections. Vaginal ultrasonography ruled out uterine pyometra and myoma of the cervix. Concerning genital infections, the serological and PCR tests did not indicate *Neisseria gonorrhoeae*, *Chlamydia trachomatis*, and *Treponema pallidum*; however, her symptoms did not exclude genital infection derived from HSV infection. Eventually, cytosmear and biopsy revealed HSV infection rather than malignancy.

A search of the PubMed database revealed only three reports in the English-language literature of HSV-related cervicitis with enlarged cervix. Table 1 summarizes the three cases [4-6]. The characteristic symptoms were fever, malaise, pelvic pain, dysuria, and vaginal bleeding. Case 1, diagnosed with a serological examination, was relieved spontaneously, while Case 2, diagnosed with the biopsy of the cervix and culture, was treated with oral valacyclovir for 10 days. Case 3, diagnosed with a serological examination, was administered acyclovir for seven days as acute therapy, followed by 6 weeks as suppressive therapy. Our case spontaneously improved and had characteristic symptoms similar to the three cases reported previously. HSV type 1 infection was diagnosed with IHC of cervical biopsy. When differentiating HSV-induced cervicitis with cervical thickness from cervical cancer, it is essential to search for characteristic symptoms of HSV infection after excluding other genital infections. Even if the cervical thickness suggests symptomatic cervical cancer, imaging findings to support symptoms should be available.

**Table 1:** Characteristics of previously reported cases of herpes simplex virus (HSV) cervicitis.

Authors	Age (years)	Onset	Main symptoms	Skin rash	HSV type	Diagnostic tool	Treatment
Tomkins et al. [4]	35	New	Dysuria	Blisters	N/A	Serum antibodies	None
Clure et al. [5]	18	New	Watery discharge	None	Type 2	Biopsy and culture	Valacyclovir
Boldrini et al. [6]	20	New	Pelvic pain, abnormal vaginal discharge	None	Type 2	Serum antibodies	Acyclovir

Appropriate diagnosis of genital herpes is important as HSV can cause serious complications, especially for women of childbearing age or immunocompromised patients. HSV rarely causes encephalitis with serious sequelae involving becoming bedridden, cognitive impairment, and memory impairment in HIV-infected people and neonates [2,5]. Regarding HSV types, HSV type 1 tends to develop serious complications such as encephalitis and is associated with a poor prognosis, while the recurrence of HSV type 1 is less likely within a year compared with HSV type 2 [9-11]. Hence, distinguishing between HSV type 1 and 2 infections is important in estimating prognosis. Additionally, HSV infection among women of childbearing age or pregnant women is a threat. The transmission rates from the mother to the fetus for primary infection and reinfection are reported to be 30%-60% and 0%-3%, respectively [12]. Neonatal herpes is classified into three types, namely, skin, eye, or mucous membrane disease; central nervous system disease; and disseminated disease with mortality rates of 0%, 14%, and 29%, respectively, even if acyclovir is administered [13]. Nevertheless, in pregnant women with primary infection, the administration of acyclovir is recommended as it can reduce viral exposure to the fetus and shorten the duration of illness, thereby decreasing the rate of cesarean section and in utero infection, although the effectiveness of acyclovir is unclear in pregnant women with reinfection [14]. An appropriate diagnosis of genital herpes, which may lead to a reduction of neonatal death, is also important in preventing HSV infection in neonates for women of childbearing age or pregnant women. The administration of antiviral drugs remains a subject of debate; however, early diagnosis could lead to timely treatment and a potential reduction in long-term complications. Hence, considering HSV as a differential diagnosis for cervical swelling is crucial.

## Conclusions

We described a rare case of HSV type 1 infection with prominent cervical swelling. Symptoms such as fever, abdominal pain, and dysuria and imaging findings are key to differentiating cervical cancer from HSV infection. Furthermore, the diagnosis of genital herpes, including HSV, is essential for women of childbearing age from the perspective of prognosis and neonatal herpes. Considering herpes-related cervicitis in the differential diagnosis for cervical swelling and achieving an early diagnosis may help reduce the risk of long-term complications.

## References

[1] Whitley RJ, Roizman B (2001). Herpes simplex virus infections. Lancet.

[2] Karasneh GA, Shukla D (2011). Herpes simplex virus infects most cell types in vitro: clues to its success. Virol J.

[3] Gupta R, Warren T, Wald A (2007). Genital herpes. Lancet.

[4] Tomkins A, White C, Higgins SP (2015). Primary herpes simplex virus infection mimicking cervical cancer. BMJ Case Rep.

[5] Clure C, Rivard C (2018). Primary herpes simplex virus infection mimicking a cervical malignancy in an immunocompetent individual. Cureus.

[6] Boldrini L P, Vallejos P G, Ballesteros P P, Valenzuela L G, Roncone D E (2023). Tumor-like presentation of herpetic cervicitis: a case report. Case Rep Womens Health.

[7] Lashmanova N, Braun A, Cheng L, Gattuso P, Yan L (2022). Endocervical adenocarcinoma in situ-from Papanicolaou test to hysterectomy: a series of 74 cases. J Am Soc Cytopathol.

[8] Khosla D, Gupta R, Srinivasan R, Patel FD, Rajwanshi A (2012). Sarcomas of uterine cervix: clinicopathological features, treatment, and outcome. Int J Gynecol Cancer.

[9] Lee GH, Kim J, Kim HW, Cho JW (2021). Herpes simplex viruses (1 and 2) and varicella-zoster virus infections in an adult population with aseptic meningitis or encephalitis: a nine-year retrospective clinical study. Medicine (Baltimore).

[10] Steiner I, Kennedy PG, Pachner AR (2007). The neurotropic herpes viruses: herpes simplex and varicella-zoster. Lancet Neurol.

[11] Sauerbrei A (2016). Optimal management of genital herpes: current perspectives. Infect Drug Resist.

[12] (2007). ACOG Practice Bulletin. Clinical management guidelines for obstetrician-gynecologists. No. 82 June 2007. Management of herpes in pregnancy. Obstet Gynecol.

[13] Kimberlin DW, Lin CY, Jacobs RF (2001). Safety and efficacy of high-dose intravenous acyclovir in the management of neonatal herpes simplex virus infections. Pediatrics.

[14] Pinninti SG, Angara R, Feja KN (2012). Neonatal herpes disease following maternal antenatal antiviral suppressive therapy: a multicenter case series. J Pediatr.

